# Current Attitudes of Chinese Dairy Practitioners to Pain and Its Management in Intensively Raised Dairy Cattle

**DOI:** 10.3390/ani12223140

**Published:** 2022-11-14

**Authors:** Ruijia Shi, Hang Shu, Ruyang Yu, Yajing Wang, Ziqi Zhang, Junjie Zhang, Xianhong Gu

**Affiliations:** 1 State Key Laboratory of Animal Nutrition, Institute of Animal Sciences, Chinese Academy of Agricultural Sciences, Beijing 100193, China; 2 College of Veterinary Medicine, China Agricultural University, Beijing 100193, China; 3 State Key Laboratory of Animal Nutrition, College of Animal Science and Technology, China Agricultural University, Beijing 100193, China; 4 Boehringer Ingelheim Animal Health (Shanghai) Co., Ltd., Shanghai 200040, China

**Keywords:** animal welfare, intensive farming, questionnaire survey, attitude

## Abstract

**Simple Summary:**

No previous studies have focused on pain perception and management in intensively raised dairy cattle in China. The results of this survey study show that severely painful conditions such as parturition and fracture have the highest score in pain perception. Pain perception is highly associated with pain management willingness. To promote animal welfare and reduce unnecessary production losses, training in pain perception and management should be emphasised.

**Abstract:**

Pain in dairy cattle is gaining attention globally. This study investigated the current attitudes of Chinese dairy practitioners to pain and its management in intensively raised dairy cattle. A total of 465 valid questionnaires with 26 painful conditions scored on numerical rating scales were collected from dairy practitioners. Data were analysed by descriptive statistics, analysis of variance, principal component analysis, and multivariate regression models. Dystocia was perceived as the most painful, while mild mastitis with milk changes only was perceived as the least painful. Respondents who agreed with the statement “pain management is worthwhile” tended to give a higher pain score. Young respondents (≤23 years old) and those from farms with ≤1000 cattle had lower pain scores for conditions with severe pain and low variability but higher pain scores for conditions with less severe pain and high variability, whereas highly educated respondents had consistently lower pain scores. As for pain management, older respondents (≥24 years old) tended to choose non-steroidal anti-inflammatory drugs, and farms with >1000 cattle were more likely to use analgesics. Training in pain perception and management should be emphasised with the hope of promoting animal welfare and reducing unnecessary production losses.

## 1. Introduction

Pain is one of the most common clinical signs that can be suffered physiologically and psychologically. With research advances in neurophysiology, the fact that nonhuman animals without verbal ability experience pain is increasingly recognised [1]. Thus, reducing pain in animals through prevention and treatment is urgently advocated [2].

In modern intensive housing systems, many conditions (e.g., mastitis and dystocia) and management procedures (e.g., horn removal) can cause varying degrees of pain to dairy cattle [3]. Generally, pain can be assessed through objective and subjective methods. The former mainly includes blood biomarkers, while the latter is usually conducted by farmers or veterinarians based on their observations of animal behaviours [4]. Of note, objective measurements are expensive and can cause stress in animals. As a result, subjective perception is still dominant in regular pain assessment in practice.

The use of pain management varies across countries. In New Zealand, local anaesthetic must be used before horn removal since October 2019 [5]. In the UK and Canada, a few non-steroidal anti-inflammatory drugs (NSAIDs) have been approved for the treatment of pain in livestock species [6]. In the United States, pain management has not been widely used in the cattle industry due to the strict regulation of NSAIDs [7]. In China, there is currently no clear guidance on analgesic use regarding pain management in cattle. It is, therefore, unknown whether people are making appropriate analgesic choices.

Many factors (e.g., gender, education level, decade of graduation) have been identified to have an impact on people’s attitudes toward cattle pain and its management in relevant investigations conducted all over the world [8,9]. However, there is no report on pain perception and management in Chinese dairy cows. The dairy industry in China has been transforming from small-scale traditional systems to large-scale intensive systems over the past decades [10]. The increasing trend of intensive farming coupled with the lack of appropriate pain perception and management may lead to animal welfare problems. It is, therefore, necessary to investigate Chinese dairy practitioners’ attitudes towards pain and its management in dairy cattle and to what extent previously identified factors affect their attitudes.

Therefore, this preliminary cross-sectional study focused on Chinese intensive farming systems, intending to investigate (1) how dairy practitioners perceive pain in cattle and (2) how they use analgesics for pain management.

## 2. Materials and Methods

### 2.1. Survey and Questionnaire Design

The target population of this questionnaire survey was Chinese dairy practitioners, including frontline staff (feeding/breeding/milking staff), managers, and veterinarians who serve in an intensive dairy system. The questionnaire was designed in three sections with a total of 10 questions using the Wenjuanxing system (https://wjx.cn/, a popular online survey site in China) accessed on 1 March 2021. The first section consisted of four demographic questions, including gender, age, education level, and position, as well as three farm-related questions, including the farming strategy (intensive or extensive), farm scale, and farm address. In the second section, 26 conditions that were considered painful for dairy cattle [8,9] were given with descriptions when applicable (see Table A1), and respondents were asked to score the severity of pain under each condition on a numerical rating scale of 0 (no pain at all) to 10 (worst pain) [11]. The scoring assumed no use of pain management. Clinical mastitis was classified into mild, moderate, and severe conditions as per Oliveira et al. [12], with descriptions given as follows: “only the milk is abnormal”, “abnormal milk and swelling or redness of mammary gland”, and “systemic signs such as depression, anorexia, dehydration, or fever”, respectively. To avoid potential measurement bias caused by inappropriate use of the pain scale, an illustrative guide with an example was given. The third section included a question about whether they agreed with the statement “pain management is worthwhile” for each condition, and a multiple-response question to choose which kind(s) of analgesics they would use from three options: local anaesthetics, NSAIDs, and sedatives.

The questions were reviewed by collaborating professionals specialising in veterinary medicine and animal welfare. The online questionnaire was piloted by eight dairy practitioners from intensive farms to ensure its feasibility and validity. No formal sample size calculation was performed, and a convenience sample of participants was used for this survey. On 31 March 2021, the advertisement for the questionnaire was issued on four relevant WeChat public accounts with good reach to Chinese dairy practitioners for a period of 1 month. Anonymity was guaranteed, and gratitude was kindly expressed to the respondents. Within 1 month, 2761 people checked and read the questionnaire, of which 666 people completed and submitted it.

### 2.2. Data Processing and Analysis

Data were stored and processed in the spreadsheet (Excel 2016, Microsoft, Redmond, Washington, DC, USA) to summarise descriptive statistics. Respondents who did not work in an intensive dairy system were excluded from further analysis. The overall mean and standard deviation (SD) of the pain scores rated by the respondents were calculated for each condition. The answer to the multiple-response question was summarised as the proportion of the total respondents.

Further statistical analyses were performed using SAS version 9.4 (SAS Institute, Cary, NC, USA). Analyses of variance were performed using the PROC ANOVA to compare the pain scores for each condition between respondents who agreed and disagreed with the statement “pain management is worthwhile”. Principal component analysis with varimax rotation was performed using the PROC FACTOR to explore how the conditions were associated with each other in terms of their pain scores. Kaiser criterion (eigenvalue > 1) was used to extract principal components, supported by the visual assessment of the scree plot. The conditions were then merged into certain categories according to their contributions in the rotated component matrix. The PROC GLM was used to analyse the effects of gender, age, education level, position, and farm scale on the pain score of each condition category. Pairwise comparisons were performed using the Bonferroni method, and results were presented as least squares means ± standard error. To explore the factors affecting the choice of pain management, logistics regressions were performed using the PROC LOGISTIC on the three dichotomous outcomes: choose (1) or not choose (0) local anaesthetics, NSAIDs, and sedatives, respectively. Significance was declared at *p* < 0.05.

## 3. Results

### 3.1. Frequency Distribution of Respondents

**Figure 1:**
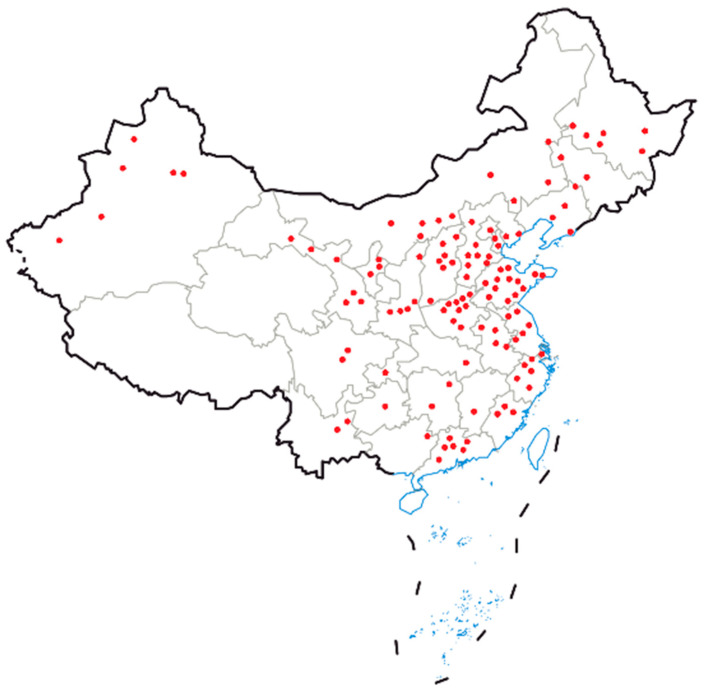
Geographical distribution of the responding dairy farms across China. Each red dot represents a city containing at least one responding dairy farm.

The response rate of people who finished their questionnaire was 24.1% (*n* = 666). The following statistical analysis used data from 465 respondents after excluding respondents who did not work in an intensive dairy system. The distribution of the respondents is shown in Table 1. According to their reported farm address, the respondents came from 399 dairy farms that were widely distributed in 30 out of 34 provincial-level divisions (Figure 1). Moreover, these farms accounted for 17.3% of the total national dairy cattle population in 2019 [13].

### 3.2. Pain Score of 26 Painful Conditions

Twenty-six painful conditions, as listed in Table 2, were investigated by pain perception scoring (range 0 to 10) and categorised as “obstetrics”, “internal medicine”, “horn removal”, “udder”, “metabolic and nutritional disease”, and “other”. Dystocia, caesarean section, calving, and fracture were ranked the top four painful conditions with mean ± SD of 9.0 ± 1.83, 8.6 ± 2.12, 8.5 ± 1.99, and 8.4 ± 2.08, respectively. The two least painful conditions were nutritional deficiency disease (3.6 ± 2.80) and mild mastitis (3.4 ± 2.65). Among three horn removal conditions, the mean pain score for calf disbudding (hot iron) was the highest but with the smallest variation (7.6 ± 2.32). The same trend was shared by three clinical mastitis conditions allocated in the “udder” category, among which severe mastitis had the highest score with the smallest variation (7.0 ± 2.20).

**Figure 2:**
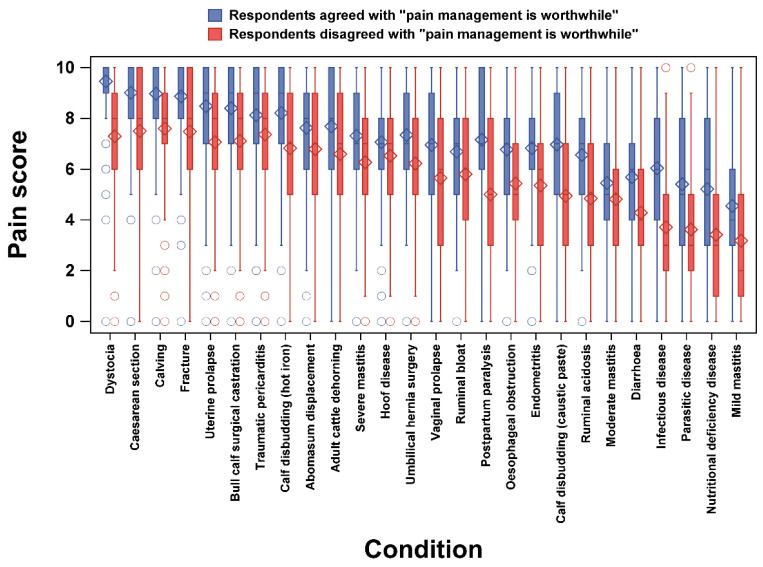
Pain scores given by respondents who agreed and disagreed with the statement “pain management is worthwhile” among 26 painful conditions. Diamonds and circles indicate means and outliers (out of 1.5 intra-quartile range), respectively.

Among 26 painful conditions, respondents who agreed with the statement “pain management is worthwhile” gave significantly higher (all *p* < 0.05) pain scores than those who disagreed, in which the smallest and largest differences were from hoof disease (difference in means: 0.5) and infectious disease (difference in means: 2.3), respectively (Figure 2).

### 3.3. Categorisation of 26 Painful Conditions

**Figure A1:**
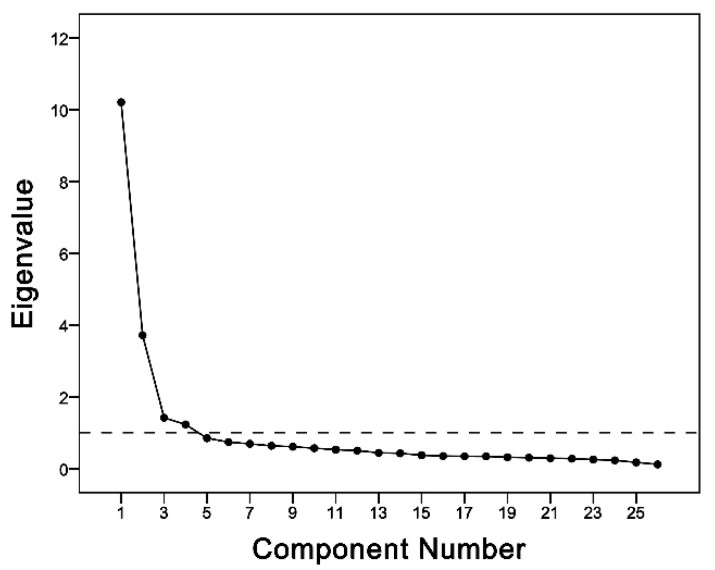
Scree plot of the principal component analysis using the pain scores of 26 painful conditions in dairy cattle scored by 465 dairy practitioners. The dashed line represents the eigenvalue of one.

The scree plot (see Figure A1) shows that four principal components had an eigenvalue higher than the mean variance of the variables (equal to one in the case of standardisation), and the eigenvalues seemed to level off since the third component. Therefore, for easier interpretation, the first two principal components were chosen, which can explain 53.6% of the total variance. The rotated component matrix is shown in Table 3, in which data with a correlation coefficient less than 0.48 has been eliminated.

The 13 conditions that correlated most to the first principal component had a higher mean with generally lower SD pain scores compared with the remaining 13 conditions that correlated most to the second principal component (see Table 2). As a result, the conditions were merged into two condition categories, with the first category characterised as severe pain with low variability and the second category characterised as less severe pain with high variability.

### 3.4. Factors Influencing Pain Perception

The pain score of two condition categories summarised by five influencing factors is shown in Table 4. For the first condition category, respondents aged ≤23 years had significantly the lowest pain scores among age groups (all *p* < 0.001); respondents with a master’s degree or above had significantly the lowest pain scores among education groups (all *p* < 0.001); respondents from a farm stocking ≤1000 cattle had significantly lower pain scores than those from a farm stocking >1000 cattle (all *p* < 0.05); there were no significant differences in pain scores given by respondents of different genders or positions (all *p* > 0.05).

For the second condition category, female respondents had significantly higher pain scores compared with male respondents (*p* < 0.001); respondents aged ≤30 years had significantly the highest pain scores among age groups (all *p* < 0.05); respondents with a bachelor’s degree had significantly higher pain scores than respondents without a college degree (*p* < 0.001), and respondents with a master’s degree or above were significantly the lowest in their pain scores among education groups (both *p* < 0.05); managers or above had significantly higher pain scores than frontline staff (*p* < 0.01), and veterinarians had significantly the lowest pain scores among position groups (both *p* < 0.01); respondents serving a farm stocking ≤1000 cattle had significantly higher pain scores than respondents serving a farm stocking >1000 cattle (all *p* < 0.01).

### 3.5. Choice of Analgesics

**Figure 3:**
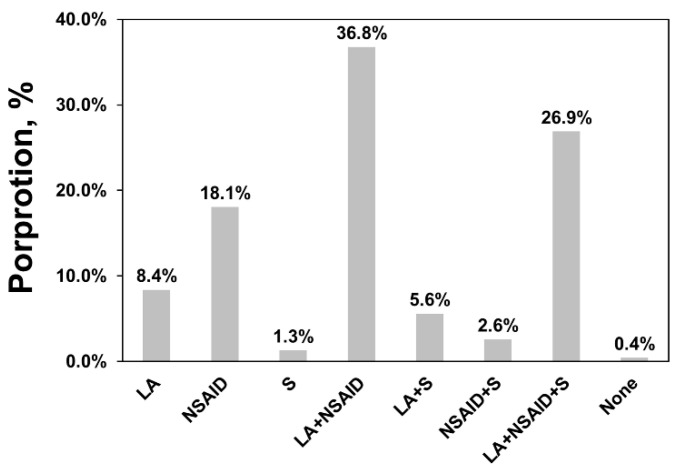
Summary of the answer to the multiple-response question asking the respondents to choose which kind(s) of analgesics they would use from three options: local anaesthetic (LA), non-steroidal anti-inflammatory drug (NSAID), and sedative (S).

Local anaesthetics plus NSAIDs was the most popular answer for pain management (36.8%), followed by a combination of all three analgesics (26.9%) (Figure 3). When summarising three choices separately, most respondents chose NSAIDs (84.3%), followed by local anaesthetics (77.6%) and sedatives (36.3%).

### 3.6. Factors Influencing the Choice of Analgesics

**Figure 4:**
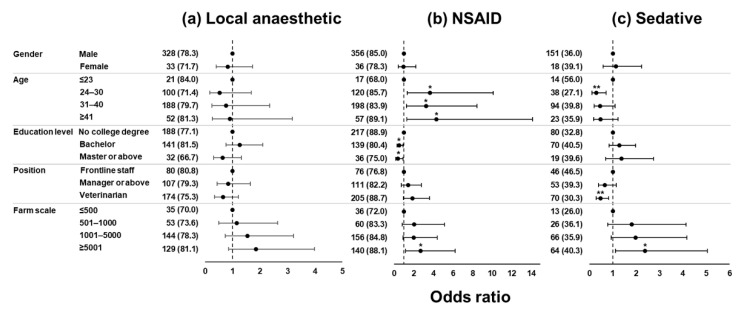
Forest plot showing the association between demographics and farm-related characteristics and the choice of (**a**) local anaesthetic, (**b**) non-steroidal anti-inflammatory drug (NSAID), and (**c**) sedative. The respondents choosing each pain management were summarised as actual number (*n*) and proportion of each sub-category (%). The dashed lines represent an odds ratio of one. * and ** indicate statistical significance (*p* < 0.05 and *p* < 0.01, respectively).

The forest plot showing the results of logistic regression models for the choice of three analgesics is presented in Figure 4. Male, aged ≤ 23 years, no college degree, frontline staff, and farms with ≤500 cattle were treated as the reference for gender, age, education level, position, and farm scale, respectively.

For the use of local anaesthetics, the 95% confidence intervals of odds ratio (OR) all included one, indicating no significant difference (all *p* > 0.05). For the use of NSAIDs, respondents aged over 24 years were more likely to choose NSAIDs (OR = 3.65, 3.24, and 4.30 for age groups of 24 to 30 years, 31 to 40 years, and ≥41 years, respectively; all *p* < 0.05); respondents with a bachelor’s degree or more were less likely to use NSAIDs (OR = 0.50 and 0.39 for education levels of bachelor and master or more, respectively, both *p* < 0.05); respondents serving a farm stocking ≥5001 cattle were more likely to use NSAIDs (OR = 2.69, *p* = 0.02). For the use of sedatives, respondents aged 24 to 30 years and veterinarians were less likely to use sedatives (OR = 0.29 and 0.49, respectively, both *p* < 0.01), whereas respondents serving a farm stocking ≥5001 cattle were more likely to use sedatives (OR = 2.38, *p* = 0.02).

## 4. Discussion

### 4.1. Pain Score of 26 Painful Conditions

It is necessary to accurately assess the severity of pain that cattle suffer from various diseases and procedures to inform pain management decisions on dairy farms. Subjective pain perception still provides valuable information until more advanced artificial intelligence-based objective methods are available for massive measurements. To the best of our knowledge, this was the first investigation of pain perception and analgesic use for intensively farmed dairy cattle in China. Such a survey allows the quantification of how perceived pain deviates from previous knowledge as well as from the pain identified by other studies which were conducted in other countries. In this study, conditions in the “obstetrics” category (i.e., dystocia, caesarean section, and calving) and fracture were considered the most painful, whereas mild mastitis with milk changes only was considered the least painful. These results are consistent with previous studies conducted in other countries [9,14,15], suggesting that Chinese dairy practitioners have relevant attitudes toward pain in cattle.

Caustic paste disbudding was perceived to have a lower level of pain compared with hot iron disbudding. This result is consistent with a recent survey in which caustic paste was perceived as the least painful instrument by Brazilian animal scientists and veterinarians [16]. According to our unpublished data, more than 90% of the dairy farms in China use caustic paste as their primary disbudding method. This proportion is dramatically higher than that in the UK [17], Europe [18], and North America [19]. It should be noted that most studies and recommendations suggest hot iron as a preferable method for disbudding since it induces less total plasma cortisol responses [20] and less negative impact on calves’ behaviours [21]. Given that caustic paste causes less response during application but more postoperative pain, the lower level of pain perceived by dairy practitioners in this study might be biased by the lack of postoperative observation [20].

An interesting trend identified in this study is that the more severe the pain condition (e.g., severe mastitis), the smaller the variation in the perceived score; the less severe the pain condition (e.g., mild mastitis), the greater the variation in the perceived score. This can be easily explained by the fact that severe conditions have more noticeable clinical signs than mild and moderate conditions [22] and therefore are more likely to be scored consistently. Moreover, cattle’s nature to hide their vulnerability to better compete for resources and avoid being attacked by predators [4] probably makes less painful conditions even more difficult to identify. However, the pain caused by mild to moderate diseases, which is less perceivable by people, actually happens more frequently than that caused by severe diseases [23]. Therefore, the large variation in perceived pain for less severe conditions suggests that there is still room for improvement in pain perception so that certain diseases (e.g., mastitis) can be better detected and treated in their early stage.

Our results show that the pain scores given by respondents who disagreed with the statement “pain management is worthwhile” were lower than those given by respondents who agreed. In the study of Huxley and Whay [8], veterinarians who did not use any analgesia assigned significantly lower pain scores to common conditions, including dystocia, surgical castration, and umbilical hernia surgery compared with those who used analgesia. Collectively, we suggest that a lack of awareness or inability to accurately assess the level of pain experienced by cows will hinder proper analgesic management. Dairy practitioners’ perception of pain in dairy cattle as a basis for good pain management should be better understood and emphasised in practice.

### 4.2. Factors Influencing Pain Perception

Many factors can affect the level of pain perceived by dairy practitioners. Gender has been identified as an influencing factor in pain perception, with females having higher pain scores than males [8,9]. In this study, this trend was found only when perceiving pain for the second condition category, which has been characterised by less severe pain. It is well known that females perceive pain differently from males and are more animal-friendly [16]. This may be because females generally care more about others and are more likely to generate strong empathy for animals [24]. On the contrary, no gender difference was found in the attitudes and empathy toward dairy cattle welfare indicators in a survey of Norwegian dairy farmers [15]. The authors attributed their result to the significantly lower number of females.

The age of respondents affected their perceived pain scores differently for two condition categories. For the first category, which has been characterised by severe pain, younger respondents tended to give lower pain scores. This is consistent with the study of Kielland, Skjerve, Østerås, and Zanella [15], where older farmers gave the highest pain score for 21 painful conditions in dairy cattle. An opposite trend was found for the second category, where older respondents tended to give lower pain scores. This might be because their richer hands-on experiences make them believe that some pains are beneficial in preventing excessive physical activity [25].

Lower pain scores were perceived by high education level respondents in this study, which is consistent with a survey on farmers’ perception of pain in sheep [26]. These could be explained by the fact that highly educated practitioners have less chance to be directly involved in actual practice compared with those with lower education levels who joined the farms earlier. Indeed, self-education in practice has been identified as the major source of gaining knowledge in pain recognition [25]. Our result may reveal a deficiency of appropriate training on pain perception in college. Relevant training should be strengthened, especially for students who are more likely to engage in actual production after graduation.

Our results also indicate that veterinarians perceived lower pain scores for conditions with less severe pain. This finding is consistent with the study of Thomsen et al. [27], in which veterinarians generally perceived lower pain scores than farmers. The difference in pain scores among positions might be due to their disparate duties on the farm. It is not doubtful that veterinarians have much more practical experience in dealing with painful conditions compared with other frontline staff and managers.

The scale of dairy farms is closely related to their economic basis, barn facilities, and technical support. Since larger farms permit more specialised and standardised management in relation to disease prevention, treatment, and pain management [28], we speculate that the technical level of dairy farms with a stock of ≤1000 cattle may need to be improved due to their lower pain scoring for severely painful conditions coupled with higher pain scoring for less painful conditions. In this case, the focus should be shifted from conditions with less severe pain to those with severe pain when taking limited resources into consideration.

Collectively, our results suggest that specialised professional training is warranted to disseminate up-to-date knowledge of cattle pain [2,9]. The perceived pain scores reported herein can be used as a reference by practitioners to identify whether they underestimate or overestimate certain pains in cattle. Moreover, recommendations from public and private extension programs should be leveraged since they have been identified to play an important role in affecting farmers’ attitudes toward dehorning practices [29].

### 4.3. Choice of Analgesics and Its Influencing Factors

The three investigated analgesics represent the most common clinical practice. Different analgesics have inconsistent analgesic effects on different types of pain, among which local anaesthetics and sedatives have relatively short-acting analgesic effects, while NSAIDs are more effective in suppressing long-lasting pain [30]. Local anaesthetic techniques are used extensively in farm animals for a variety of painful conditions, such as castration [31] and horn removal [32]. In these cases, NSAIDs are often used concomitantly to control long-lasting postoperative pain. Our results show a dominant choice of concomitant medication of local anaesthetics and NSAIDs, whereas only two respondents did not choose any pain management, indicating good awareness and willingness to provide analgesia.

In this study, NSAIDs were the most popular among 465 respondents, either in combination with other drugs or alone. The choice of NSAIDs became less popular as education level increased, which can be recognised by their lower pain scores. This fact re-emphasises the importance of appropriate pain perception. On the other hand, sedatives were the least popular answer, with the majority of use being concomitant with local anaesthetics and NSAIDs. Veterinarians were only half as likely to choose sedatives as other frontline staff. These facts may be because sedatives are advised to be used with caution, especially in pregnant animals [33]. Veterinarians, particularly those from obstetrics, are less likely to use sedatives.

The scale of dairy farms was found to be closely related to the willingness for pain management. Larger farms were more likely to use the three investigated analgesics, which are supposed to be associated with sufficient resources in professionals and finance. Cost has been identified in previous studies as an important influencing factor in the decision to use an analgesic [3]. Moreover, the least use of pain management in farms with ≤1000 cattle may be explained by their improper pain perception. In any case, our results suggest that pain perception and management in farms with ≤1000 cattle need to be improved, although the cost may be the biggest barrier.

### 4.4. Strengths and Limitations

Our results uncover the current attitudes of Chinese dairy practitioners to pain and its management in intensively raised dairy cattle and highlight the disparity among respondents with different characteristics and among farms with different scales. Consistency was found between perceived pain scores and analgesic use, demonstrating the importance of appropriate pain perception. Therefore, in addition to targeted policies against resource inequalities, professional training in pain perception shall be done first to better perceive pain in dairy cattle on farms with ≤1000 cattle.

Convenience sampling used in this survey might reduce the representation of the study base. However, this effect should have been minimised since our results show a broad geographical distribution of the responding farms as well as a sizable proportion of the dairy cattle population. Additionally, the questionnaire was distributed online and relied totally on people’s subjective wishes, which could introduce selection bias since dairy practitioners who were more interested in pain management might be more likely to participate in the survey. This bias would lead to an increased overall pain score and should be non-differential among sub-categories of respondents. It should also be noted that the analgesic use investigated in this study is the overall situation. Different analgesics are needed for different types of pain. However, detailed questions on the analgesic use for each condition were not included in the questionnaire and, therefore, could not be further analysed.

## 5. Conclusions

Chinese dairy practitioners have various attitudes to pain in dairy cattle. Severely painful conditions, such as obstetrics conditions (i.e., dystocia, caesarean section, and calving) and fracture, were perceived to have more consistent pain scores compared with less severe conditions such as mild mastitis. Perceived pain scores were highly associated with willingness to provide pain management. Thus, training in pain perception should be emphasised with the hope of promoting animal welfare and reducing unnecessary production losses. Although such survey data is subjective, our results provide insight into the current situation of pain perception and analgesic use in intensive dairy systems. The results of this study can be used as a reference by practitioners to assess their attitudes and perceptions of cattle pain and its management in practice.

## Figures and Tables

**Table 1:** The distribution of 465 respondents summarised by farm scale and presented as actual number (*n*) and proportion of each sub-category (%).

Respondents’ Characteristics	Dairy Stock of the Farms they Served				Total
	≤500	501–1000	1001–5000	≥5001	
Gender					
Male	37 (74.0)	63 (87.5)	171 (92.9)	148 (93.1)	419 (90.1)
Female	13 (26.0)	9 (12.5)	13 (7.1)	11 (6.9)	46 (9.9)
Age					
≤23	2 (4.0)	6 (8.3)	6 (3.3)	11 (6.9)	25 (5.4)
24–30	17 (34.0)	20 (27.8)	56 (30.4)	47 (29.6)	140 (30.1)
31–40	23 (46.0)	35 (48.6)	93 (50.5)	85 (53.5)	236 (50.8)
≥41	8 (16.0)	11 (15.3)	29 (15.8)	16 (10.1)	64 (13.8)
Education level					
No college degree	29 (58.0)	33 (45.8)	93 (50.5)	89 (56.0)	244 (52.5)
Bachelor	12 (24.0)	30 (41.7)	73 (39.7)	58 (36.5)	173 (37.2)
Master or above	9 (18.0)	9 (12.5)	18 (9.8)	12 (7.5)	48 (10.3)
Position					
Frontline staff ^1^	22 (44.0)	16 (22.2)	32 (17.4)	29 (18.2)	99 (21.3)
Manager or above	15 (30.0)	22 (30.6)	57 (31.0)	41 (25.8)	135 (29.0)
Veterinarian	13 (26.0)	34 (47.2)	95 (51.6)	89 (56.0)	231 (49.7)
Total	50 (100.0)	72 (100.0)	184 (100.0)	159 (100.0)	465 (100.0)

^1^ Feeding/breeding/milking staff.

**Table 2:** Pain score (range 0–10) of the 26 painful conditions in adult dairy cattle and calves scored by 465 dairy practitioners.

Category	Condition	Mean	SD
Obstetrics	Dystocia	9.0	1.83
	Caesarean section	8.6	2.12
	Calving	8.5	1.99
	Uterine prolapse	7.9	2.30
	Vaginal prolapse	6.3	2.66
	Endometritis	5.9	2.62
Internal medicine	Traumatic pericarditis	7.8	2.25
	Abomasum displacement	7.4	2.18
	Ruminal bloat	6.1	2.40
	Oesophageal obstruction	5.9	2.36
	Ruminal acidosis	5.3	2.62
Horn removal	Calf disbudding (hot iron)	7.6	2.32
	Adult cattle dehorning	7.1	2.52
	Calf disbudding (caustic paste)	5.6	2.74
Udder	Severe mastitis	7.0	2.20
	Moderate mastitis	5.1	2.24
	Mild mastitis	3.4	2.65
Metabolic and nutritional disease	Postpartum paralysis	5.9	3.02
	Nutritional deficiency disease	3.6	2.80
Other	Fracture	8.4	2.08
	Bull calf surgical castration	7.8	2.32
	Hoof disease	6.9	2.18
	Umbilical hernia surgery	6.8	2.35
	Diarrhoea	4.6	2.50
	Infectious disease	4.5	2.91
	Parasitic disease	4.1	2.57

SD = standard deviation.

**Table 3:** Rotated component matrix for the first two principal components after eliminating the correlation coefficients less than 0.48 ^1^.

Condition	Component	
	1	2
Dystocia	0.830	
Fracture	0.817	
Caesarean section	0.814	
Bull calf surgical castration	0.758	
Calving	0.755	
Traumatic pericarditis	0.716	
Calf disbudding (hot iron)	0.707	
Uterine prolapse	0.702	
Umbilical hernia surgery	0.668	
Abomasum displacement	0.647	
Hoof disease	0.608	
Severe mastitis	0.586	
Adult cattle dehorning	0.562	
Mild mastitis		0.818
Nutritional deficiency disease		0.810
Moderate mastitis		0.779
Parasitic disease		0.757
Diarrhoea		0.752
Ruminal acidosis		0.702
Infectious disease		0.658
Endometritis		0.604
Postpartum paralysis		0.592
Oesophageal obstruction		0.581
Ruminal bloat		0.519
Calf disbudding (caustic paste)		0.485
Vaginal prolapse		0.484

^1^ Rotation was performed using the Varimax method with Kaiser normalisation.

**Table 4:** Pain score (least squares means ± standard error) of two condition categories characterised by severe pain with low variability and less severe pain with high variability, respectively.

Factor	Group	First Category	Second Category
Gender	Male	7.4 ± 0.05	5.1 ± 0.06 ^b^
	Female	7.3 ± 0.10	5.6 ± 0.12 ^a^
Age	≤23	6.8 ± 0.14 ^b^	5.7 ± 0.08 ^a^
	24–30	7.6 ± 0.07 ^a^	5.7 ± 0.08 ^a^
	31–40	7.5 ± 0.06 ^a^	5.5 ± 0.07 ^b^
	≥41	7.5 ± 0.09 ^a^	5.0 ± 0.11 ^c^
Education level	No college degree	7.7 ± 0.07 ^a^	5.3 ± 0.08 ^b^
	Bachelor	7.6 ± 0.07 ^a^	5.6 ± 0.09 ^a^
	Master or above	6.7 ± 0.10 ^b^	5.0 ± 0.12 ^c^
Position	Frontline staff ^1^	7.2 ± 0.08	5.3 ± 0.10 ^b^
	Manager or above	7.3 ± 0.08	5.7 ± 0.09 ^a^
	Veterinarian	7.3 ± 0.07	5.0 ± 0.09 ^c^
Farm scale	≤500	7.1 ± 0.10 ^b^	5.8 ± 0.12 ^a^
	501–1000	7.1 ± 0.09 ^b^	5.5 ± 0.11 ^a^
	1001–5000	7.4 ± 0.07 ^a^	5.0 ± 0.09 ^b^
	≥5001	7.4 ± 0.08 ^a^	5.0 ± 0.09 ^b^

^1^ Feeding/breeding/milking staff. ^a–c^ Values within a column of a factor with different superscripts differ significantly at *p* < 0.05.

## Data Availability

Data available on request due to restrictions, e.g., privacy or ethical.

## Appendix A

**Table A1:** Description of 26 conditions.

Condition	Description
Dystocia	Slow or difficult labor or birth
Caesarean section	Extraction of the fetus or foeti from the mother animal through a surgical opening in the abdominal wall and the uterus
Calving	A cow giving birth to a calf
Uterine prolapse	Uterus slips down into or protrudes out of the vagina
Vaginal prolapse	An abnormally positioned vagina, seen as a pink mass of tissue that protrudes outside of the cow’s body
Endometritis	An inflammation of the endometrium, often accompanied by congestion, edema, pus, or discharge
Traumatic pericarditis	A purulent inflammation caused by the pathogen brought in by sharp things such as nails that pierce the pericardium
Abomasum displacement	An abnormal position of the abomasum in the abdominal cavity
Ruminal bloat	An excessive volume of gas builds up in the rumen during fermentation that cannot escape
Oesophageal obstruction	Esophagus is blocked by food or foreign objects
Ruminal acidosis	A metabolic disorder, witnessed by a sharp decrease in rumen pH, often caused by the intake of large amounts of easily fermented carbohydrate feed
Calf disbudding (hot iron)	Horn bud growth is prevented through tissue cauterisation
Adult cattle dehorning	Removing the horns from cattle
Calf disbudding (caustic paste)	Horn tissue is cauterised by the combination of caustic substances in dehorning paste to prevent its growth
Severe mastitis	Systemic signs such as depression, anorexia, dehydration, or fever
Moderate mastitis	Abnormal milk and swelling or redness of mammary gland
Mild mastitis	Only the milk is abnormal
Postpartum paralysis	Cows cannot move normally after calving and fall to the ground, usually caused by hypocalcemia
Nutritional deficiency disease	Various symptoms caused by insufficient intake of nutrients, such as protein, vitamins, and mineral
Fracture	Complete or partial break in the continuity of bone
Bull calf surgical castration	Process of removal or destruction of the testicles
Hoof disease	Hoof cutin rot, suppuration of skin and tissue between the toes, and movement disorder
Umbilical hernia surgery	Procedure of reducible umbilical hernia in calves
Diarrhoea	Feces have high water content, become thin, and even contain undigested food, pus, and mucus, and the number of defecation increases significantly
Infectious disease	Diseases caused by bacteria, fungi, or viruses
Parasitic disease	Internal helminths (roundworms, tapeworms, and flukes) or external arthropods (mites, lice, ticks, and flies) parasitise on cattle

## References

[1] IASP (International Association for the Study of Pain) Pain Terms. https://www.iasp-pain.org/resources/terminology/#pain.

[2] Weary D.M., Niel L., Flower F.C., Fraser D. (2006). Identifying and preventing pain in animals. Appl. Anim. Behav. Sci..

[3] Steagall P.V., Bustamante H., Johnson C.B., Turner P.V. (2021). Pain Management in Farm Animals: Focus on Cattle, Sheep and Pigs. Animals.

[4] Hudson C., Whay H., Huxley J. (2008). Recognition and management of pain in cattle. Practice.

[5] Espinoza C., Lomax S., Windsor P. (2020). The Effect of Topical Anaesthesia on the Cortisol Responses of Calves Undergoing Dehorning. Animals.

[6] Johnstone E.C.S., Coetzee J.F., Pinedo P.J., Edwards-Callaway L. (2021). Current attitudes of veterinarians and producers regarding the use of local and systemic analgesia in beef and dairy cattle in the United States. J. Am. Vet. Med. Assoc..

[7] Robles I., Arruda A.G., Nixon E., Johnstone E., Wagner B., Edwards-Callaway L., Baynes R., Coetzee J., Pairis-Garcia M. (2021). Producer and Veterinarian Perspectives towards Pain Management Practices in the US Cattle Industry. Animals.

[8] Huxley J.N., Whay H.R. (2006). Current attitudes of cattle practitioners to pain and the use of analgesics in cattle. Vet. Rec..

[9] Laven R.A., Huxley J.N., Whay H.R., Stafford K.J. (2009). Results of a survey of attitudes of dairy veterinarians in New Zealand regarding painful procedures and conditions in cattle. N. Z. Vet. J..

[10] Ministry of Agriculture (2016). China Animal Husbandry Yearbook.

[11] Hawker G.A., Mian S., Kendzerska T., French M. (2011). Measures of adult pain: Visual Analog Scale for Pain (VAS Pain), Numeric Rating Scale for Pain (NRS Pain), McGill Pain Questionnaire (MPQ), Short-Form McGill Pain Questionnaire (SF-MPQ), Chronic Pain Grade Scale (CPGS), Short Form-36 Bodily Pain Scale (SF-36 BPS), and Measure of Intermittent and Constant Osteoarthritis Pain (ICOAP). Arthritis Care Res..

[12] Oliveira L., Hulland C., Ruegg P.L. (2013). Characterization of clinical mastitis occurring in cows on 50 large dairy herds in Wisconsin. J. Dairy Sci..

[13] Dairy Association of China (2020). China Dairy Yearbook (2019).

[14] Lombard J.E., Garry F.B., Tomlinson S.M., Garber L.P. (2007). Impacts of Dystocia on Health and Survival of Dairy Calves. J. Dairy Sci..

[15] Kielland C., Skjerve E., Østerås O., Zanella A.J. (2010). Dairy farmer attitudes and empathy toward animals are associated with animal welfare indicators. J. Dairy Sci..

[16] Canozzi M.E.A., Borges J.A.R., Barcellos J.O.J. (2022). Which factors can influence the perception of pain by veterinarians and animal scientists from Brazil?. J. Vet. Behav..

[17] Hambleton S.Y.N., Gibson T. (2017). A study investigating the attitudes and opinions of cattle farmers and veterinarians in the United Kingdom on the use of Non-Steroidal Anti-Inflammatory Drugs (NSAIDs) for post-disbudding analgesia of calves. Anim. Welf..

[18] Cozzi G., Gottardo F., Brscic M., Contiero B., Irrgang N., Knierim U., Pentelescu O., Windig J.J., Mirabito L., Kling Eveillard F. (2015). Dehorning of cattle in the EU Member States: A quantitative survey of the current practices. Livest. Sci..

[19] Saraceni J., Winder C.B., Renaud D.L., Miltenburg C., Nelson E., Van Os J.M.C. (2021). Disbudding and dehorning practices for preweaned dairy calves by farmers in Wisconsin, USA. J. Dairy Sci..

[20] Stafford K., Mellor D. (2011). Addressing the pain associated with disbudding and dehorning in cattle. Appl. Anim. Behav. Sci..

[21] Ede T., von Keyserlingk M.A.G., Weary D.M. (2020). Conditioned place aversion of caustic paste and hot-iron disbudding in dairy calves. J. Dairy Sci..

[22] Leslie K.E., Petersson-Wolfe C.S. (2012). Assessment and Management of Pain in Dairy Cows with Clinical Mastitis. Vet. Clin. N. Am. Food Anim. Pract..

[23] Krishnamoorthy P., Goudar A.L., Suresh K.P., Roy P. (2021). Global and countrywide prevalence of subclinical and clinical mastitis in dairy cattle and buffaloes by systematic review and meta-analysis. Res. Vet. Sci..

[24] Estévez-Moreno L.X., María G.A., Sepúlveda W.S., Villarroel M., Miranda-de la Lama G.C. (2021). Attitudes of meat consumers in Mexico and Spain about farm animal welfare: A cross-cultural study. Meat Sci..

[25] Whay H.R., Huxley J. (2005). Pain relief in cattle: A practitioners perspective. Cattle Pract..

[26] Larrondo C., Bustamante H., Gallo C. (2018). Sheep Farmers’ Perception of Welfare and Pain Associated with Routine Husbandry Practices in Chile. Animals.

[27] Thomsen P.T., Anneberg I., Herskin M.S. (2012). Differences in attitudes of farmers and veterinarians towards pain in dairy cows. Vet. J..

[28] Robbins J., Von Keyserlingk M., Fraser D., Weary D. (2016). Invited review: Farm size and animal welfare. J. Anim. Sci..

[29] Cardoso C.S., von Keyserlingk M.A.G., Hötzel M.J. (2016). Trading off animal welfare and production goals: Brazilian dairy farmers’ perspectives on calf dehorning. Livest. Sci..

[30] Coetzee H. (2017). Pharmacological approaches to pain management in cattle. J. Anim. Sci..

[31] Coetzee J. (2013). Assessment and Management of Pain Associated with Castration in Cattle. Vet. Clin. N. Am. Food Anim. Pract..

[32] Reedman C.N., Duffield T.F., DeVries T.J., Lissemore K.D., Karrow N.A., Li Z., Winder C.B. (2020). Randomized control trial assessing the efficacy of pain control strategies for caustic paste disbudding in dairy calves younger than 9 days of age. J. Dairy Sci..

[33] Hodgson D.S., Dunlop C.I., Chapman P.L., Smith J.A. (2002). Cardiopulmonary effects of xylazine and acepromazine in pregnant cows in late gestation. Am. J. Vet. Res..

